# New Specimens of *Nemegtomaia* from the Baruungoyot and Nemegt Formations (Late Cretaceous) of Mongolia

**DOI:** 10.1371/journal.pone.0031330

**Published:** 2012-02-08

**Authors:** Federico Fanti, Philip J. Currie, Demchig Badamgarav

**Affiliations:** 1 Dipartimento di Scienze della Terra e Geologico-Ambientali, Alma Mater Studiorum, Università di Bologna, Via Zamboni, Bologna, Italy; 2 Department of Biological Sciences, University of Alberta, Edmonton, Alberta, Canada; 3 Paleontological Center, Mongolian Academy of Sciences, Ulaan Baatar, Mongolia; Institut de Biologia Evolutiva - Universitat Pompeu Fabra, Spain

## Abstract

Two new specimens of the oviraptorid theropod *Nemegtomaia barsboldi* from the Nemegt Basin of southern Mongolia are described. Specimen MPC-D 107/15 was collected from the upper beds of the Baruungoyot Formation (Campanian-Maastrichtian), and is a nest of eggs with the skeleton of the assumed parent of *Nemegtomaia* on top in brooding position. Much of the skeleton was damaged by colonies of dermestid coleopterans prior to its complete burial. However, diagnostic characters are recovered from the parts preserved, including the skull, partial forelimbs (including the left hand), legs, and distal portions of both feet. *Nemegtomaia* represents the fourth known genus of oviraptorid for which individuals have been found on nests of eggs. The second new specimen, MPC-D 107/16, was collected a few kilometers to the east in basal deposits of the Nemegt Formation, and includes both hands and femora of a smaller *Nemegtomaia* individual. The two formations and their diverse fossil assemblages have been considered to represent sequential time periods and different environments, but data presented here indicate partial overlap across the Baruungoyot-Nemegt transition. All other known oviraptorids from Mongolia and China are known exclusively from xeric or semi-arid environments. However, this study documents that *Nemegtomaia* is found in both arid/aeolian (Baruungoyot Formation) and more humid/fluvial (Nemegt Formation) facies.

## Introduction

Oviraptorid dinosaurs have long been a source of information and speculation about the behavior of non-avian theropods [1], [2]. Known since the Third Central Asiatic Expedition led by Roy Chapman Andrews [3] and for decades mistakenly referred to as exemplary egg thieves, this group of edentulous maniraptoran dinosaurs is now studied for its sophisticated social structure and behavior, which includes the brooding and care of eggs in specially prepared nests [4]–[9]. The family Oviraptoridae, known to date from the Late Cretaceous of Asia where multiple complete or nearly complete skeletons have been recovered, includes two widely accepted clades, Ingeniinae and Oviraptorinae [10], [11]. Barsbold [12] established the subfamily Ingeniinae, and separated ingeniines from oviraptorines on the basis of their smaller size and reduced manual digits II and III. The clade included both *Conchoraptor* and *Ingenia*. The first detailed phylogenetic analysis of the Oviraptoridae was published by Maryańska et al. [13]. They retrieved *Conchoraptor* and *Ingenia* in a monophyletic clade defined by a scapula to humerus ratio greater than 0.7, a deltopectoral crest that extends for 40%–50% of the length of humerus, a metacarpal II to metacarpal III ratio that is less than 0.5, and a postacetabular process of the ilium that has a truncated distal end. Osmólska et al. [10] defined ingeniines as *Conchoraptor gracilis* and *Ingenia yanshini*, their most recent common ancestor, and all descendants. The three synapomorphies they used to define the subfamily are: the synsacrum includes seven to eight vertebrae; the deltopectoral crest extends for 40%–50% of the length of humerus; and the postacetabular process of the ilium has a truncated distal end. *Khaan mckennai* was included in the Ingeniinae, and is clearly similar to *Conchoraptor*. *Nemegtomaia barsboldi* [14] is unusual in that it is the only ingeniine that has a cranial crest, and is the only oviraptorid that was collected from fluvial (instead of xeric) deposits. The type specimen (MPC-D 100/2112) was collected in 1996 in southern Mongolia at the Nemegt locality (*sensu* [15]) by the Mongolian Highland International Dinosaur Project [14]. It included the skull and partial skeleton (thirteen cervical, six dorsal, eight sacral and two caudal vertebrae, left scapula, distal ends of both humeri, right radius, both ilia, proximal ends of both pubes, both ischia, and proximal end of a femur). However, characters of the forelimbs and hands that are diagnostic for determining interspecific variation [11], [16], were unknown. Furthermore, no eggs or eggshells were found in association with the specimen. The specimen was originally referred to as *Ingenia* sp. by Lü et al. [17] but a detailed revision of the specimen led to it being renamed *Nemegtia barsboldi* [14]. However, the genus had to be renamed *Nemegtomaia* when it was realized that the name was preoccupied by a freshwater ostracod from the Nemegt Formation [18], [19]. *Nemegtomaia* (“good mother of Nemegt”) refers not only to the stratigraphic and geographic location of the discovery (early Maastrichtian Nemegt Formation, Nemegt locality, southern Gobi, Mongolia) but also to the idea that oviraptorid dinosaurs were brooding, rather than stealing, eggs [4], [5], [7]–[9], [20]–[23].

**Figure 1:**
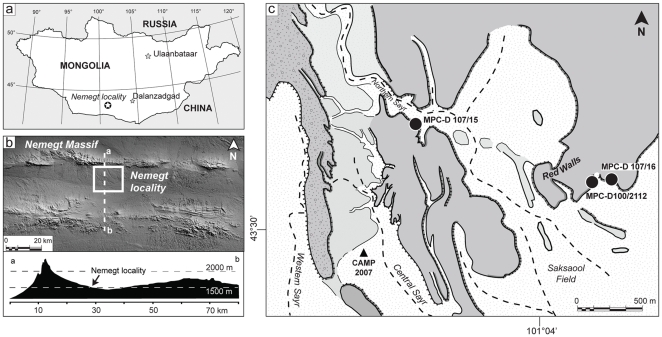
The Nemegt locality the Gobi Desert, southern Mongolia. A, map showing the location of the study area within southern Mongolia; B, the Nemegt area is located a few kilometers south of the massif of the same name; C, a detail of the Nemegt locality (*sensu* 5), showing the exact locations of specimens described in this study.

Nomadic Expedition's “Dinosaurs of the Gobi” 2007 party recovered two new specimens of oviraptorids from the Baruungoyot Formation (or “Barun Goyot” in older literature; stratigraphic terminology in this paper follows [24]) beds at the Nemegt locality (Figure 1). Specimen MPC-D 107/15 is a nest of eggs with the presumed mother lying on top. In addition to being found less than 500 m from the holotype of *Nemegtomaia*, the new specimen shares diagnostic characters of the skull and has the same absolute body size. It provides new anatomical information about the front limbs that conclusively shows that *Nemegtomaia* should be referred to the subfamily Ingeniinae. The second new specimen, MPC-D 107/16, consists of hands, ribs, a partial pelvis and both femora of a smaller individual. The two new specimens are described here with a focus on diagnostic and previously unknown characters (especially in the skull and hands), the association of the skeleton with the eggs, and the taphonomy, stratigraphic range, and paleoecology of the taxon.

### Institutional Abbreviations

AMNH, American Museum of Natural History, New York, USA; IVPP, Institute of Vertebrate Paleontology and Paleoanthropology, Beijing, People's Republic of China: MPC-D, Paleontological Center of the Mongolian Academy of Sciences, Ulaan Baatar, Mongolia ( = GIN, Geological Institute, Mongolian Academy of Sciences); UALVP, University of Alberta, Laboratory of Vertebrate Paleontology, Edmonton, Canada.

### Geology and Stratigraphy

**Figure 2:**
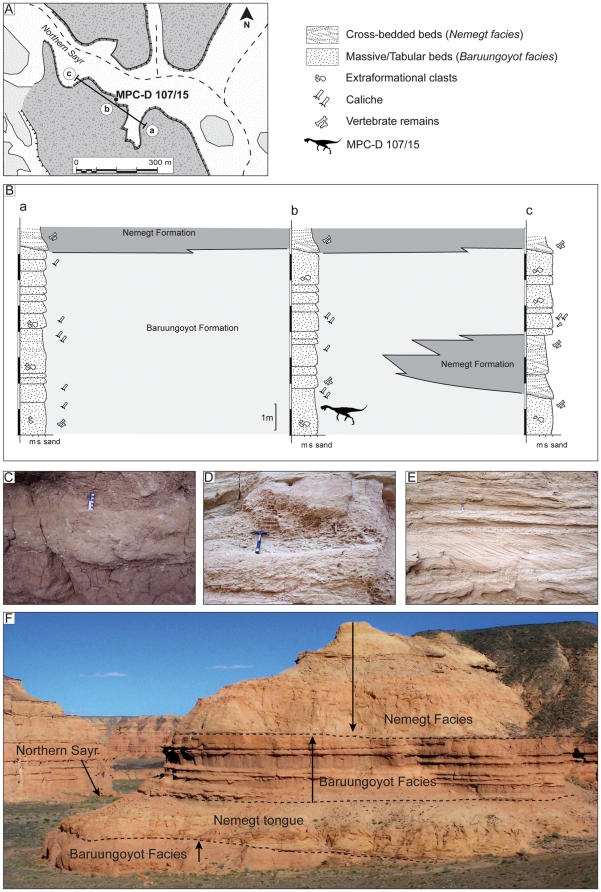
Location map showing the position of the measured section discussed in the text. A, Northern *Sayr*, where specimen MPC-D 107/15 was collected. B, lithostratigraphic logs showing the relative stratigraphic occurrence of the interfingering Baruungoyot and Nemegt formations. C, In-situ and partially reworked caliche glaebules and concretions, Baruungoyot Formation. D, tubular burrow fills interpreted as nonmarine invertebrate feeding traces, Baruungoyot Formation. E, cross-bedded deposits in the basal deposits of the Nemegt Formation. F, photograph showing the interfingering contact between the Baruungoyot and Nemegt formations in the Northern Sayr (litho-log c). MPC-D 107/15 was collected just below the Nemegt tongue deposits.

Since its discovery in 1946, the Nemegt locality (*sensu* [15]) has been recurrently investigated for the rich and diverse fossil fauna preserved in the exposed Upper Cretaceous sediments (Figure 1). Several major *sayrs* (canyons, gorges) and numerous side-canyons cut the pediment that slopes toward the south beneath the Nemegt massif. The cuts are up to 45 meters deep and provide some of the best exposures of the upper Campanian - lower Maastrichtian Baruungoyot and Nemegt formations, which have been largely discussed in the literature [25]–[33]. Specimen MPC-D 107/15 was collected from the upper Baruungoyot Formation deposits that crop out extensively along the Northern Sayr. These upper beds are barren of mudstone and consist of a stacked succession of tabular redbeds. Abundant extraformational clasts, well-developed caliches, and invertebrate feeding traces characterize these primarily aeolian deposits (Figure 2). Wedging out of single beds is fairly common, even though individual tabular beds can be traced laterally for tens or hundreds of meters. Prospecting activities carried out in Northern Sayr revealed the lateral variation of such beds, even within the cliff in which the nest was discovered (Fig. 2B). A short distance (150 meters) to the north of the nest, the distinctive Baruungoyot facies interfinger with light grey, trough cross-bedded channel deposits that are typical of the Nemegt Formation. The basal contact is sharp and erosive, juxtaposing fine- to medium-grained sandstone with a medium-grained, 5 cm thick conglomerate that fines upward into cross-bedded sandstones. Such beds form an approximately four meter thick, fining-upward tongue that is conformably overlaid by red, massive, aeolian deposits (Baruungoyot facies). Finally, the channel fill sandstone deposits of the Nemegt Formation cap this interval (Fig. 2F). The two formations and their different fossil assemblages have historically been considered to represent sequential periods of time and different environments [26], [27], [34]–[36]: the Baruungoyot Formation is indicative of semi-arid or arid environments with recurrent aeolian beds, and the Nemegt Formation has been interpreted as representing a dominantly fluvial environment with most fossils being recovered from channel fills, point bars, and some overbank deposits laid down under more humid conditions [33]. However, new data collected in the Northern Sayr at the Nemegt locality document an approximately 25 meter thick stratigraphic interval where the two formations interfinger, and support previous studies suggesting that aeolian and fluvial environments coexisted at the beginning of Nemegt sedimentation [33].

## Methods

**Figure 3:**
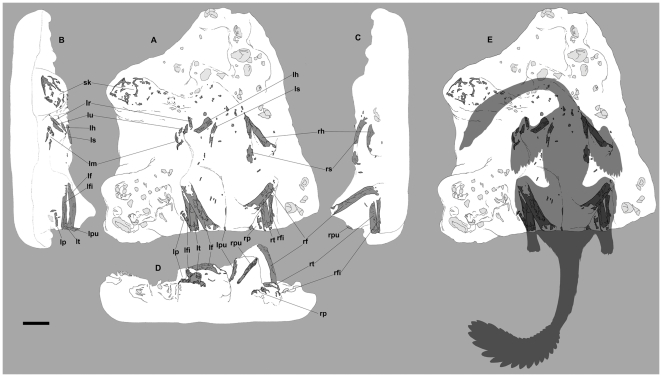
Nest and preserved elements of *Nemegtomaia barsboldi* (MPC-D 107/15). A, dorsal view; B, left lateral view; C, right lateral view; D, posterior view. **lf**, left femur; **lfi**, left fibula; **lh**, left humerus; **lm**, left manus; **lp**, left pes; **lpu**, left pubis; **lr**, left radius; **ls**, left scapula; **lt**, left tibia; **lu**, left ulna; **rf**, right femur; **rfi**, right fibula; **rh**, right humerus; **rp**, right pes; **rpu**, right pubis; **rs**, right scapula; **rt**, right tibia; **sk**, skull. Scale bar 10 cm. Reconstruction by Marco Auditore.

**Figure 4:**
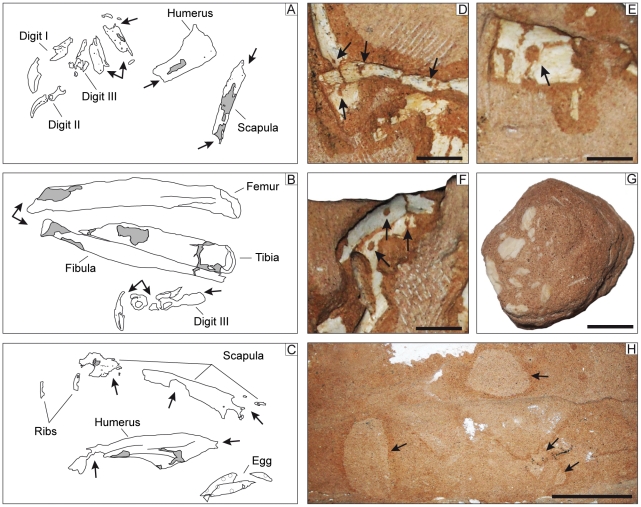
Feeding traces left by colonies of dermestid beetles in specimen MPC-D 107/15. Articular surfaces have been completely obliterated in the left forearm (A), left leg and pes (B), as well as in the right forearm (C). Circular borings are particularly evident in the left side of the skull (D–F). G, Bone-chip burrow found under the skull of the specimen. H, large traces of reworked sediment under the skeleton.

Specimen MPC-D 107/15 was collected from the red sandstones of the Baruungoyot Formation in the Northern Sayr of the Nemegt locality (N 43°30.353′, E 101°03.383′) (Figure 2). In order to preserve the spatial relationships of the bones and eggs, the specimen was collected in a single block delimited by the last occurrence of preserved eggs. The lower side of the block is only partially prepared to expose mostly egg fragments, whereas the upper surface has the articulated partial skeleton of an oviraptorid dinosaur overlying the nest (Figure 3). It includes the skull, both scapulae, the left arm and hand, right humerus, pubes, ischia, femora, tibiae, fibulae, and the distal portions of both feet. Much of the skeleton was subjected to intense scavenging by small predators and dermestid coleopterans during an early stage of burial (Figure 4).

The second specimen (MPC-D 107/16) was collected from the uppermost layers of the Baruungoyot Formation in the eastern portion of the Nemegt locality from the Red Walls (N 43°30.327′, E 101°04.706′) (Figure 1). It includes a complete right hand, the left hand with partial ulna and radius, a few ribs, both femora, and a partial ilium.

## Results

### Description of MPC-D 107/15

**Figure 5:**
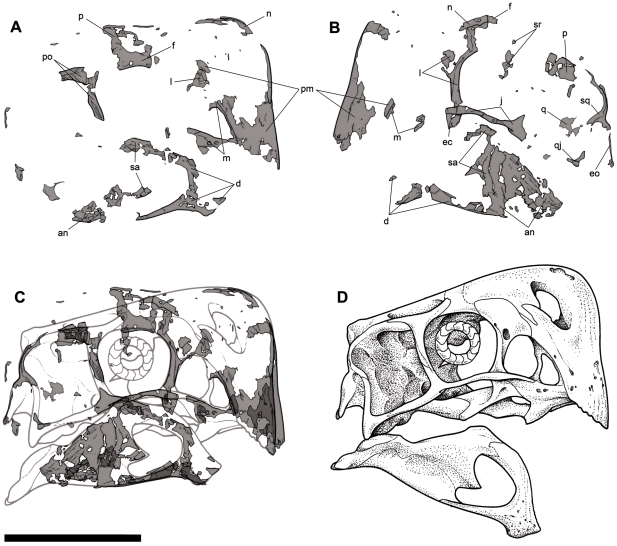
Skull of *Nemegtomaia barsboldi* (MPC-D 107/15). A, preserved elements of the right side; B, preserved elements of the left side; C, reversed elements of the left side superimposed to those of the right side (where elements of both sided overlap, the grey is darker); D, reconstruction of the skull. **an**, angular; **d**, dentary; **ec**, ectopterygoid; **eo**, exoccipital; **f**, frontal; **j**, jugal; **l**, lacrimal; **m**, maxilla; **n**, nasal; **p**, parietal; **pm**, premaxilla; **po**, postorbital; **q**, quadrate; **qj**, quadratojugal; **sa**, surangular; **sq**, squamosal; **sr**, sclerotic ring. Scale bar 10 cm. Illustration by Marco Auditore.

The oviraptorid affinities [10] of MPC-D 107/15 are unquestionable with its relatively short snout, and its deep, edentulous, beaklike lower jaws (Figure 5). Similarly, there are many characters in the postcranial skeleton as well as in the eggs that clearly show it is an oviraptorid (Figure 3). Much of MPC-D 107/15 was damaged by post-mortem, pre-burial insect activity, crushing during burial, and most recently by erosion; it is nevertheless rich in morphological and phylogenetic information. Similar to other nests of brooding oviraptorids [4], [8], [37], [38], the animal had its feet in the center of what was probably a ring of eggs, and the arms were folded across the tops of the eggs on either side of the body. Although the individual bones have been damaged by borings and other scavenging, their shapes and lengths can be reconstructed because of the fact that the bones were held immobile in the sediments while they were being eaten by insects, and because their margins are often preserved. The front of the premaxilla and the posterior margin of the paroccipital process are preserved on each side of the skull, and both sides show that the skull was 172 mm long. The dorsal and posterior margins of the orbit are preserved on the right side of the skull, whereas the anterior, ventral and ventroposterior margins of the orbit are preserved on the left side. The skull length is slightly shorter than that of the holotype of *Nemegtomaia* (179 mm), whereas the diameter of the orbit is approximately the same size (52 mm). Other overlapping measurements of the two specimens are also similar, including jaw length (153 mm in holotype, 152 mm in MPC-D 107/15), and maximum dentary height (49 mm in holotype, 50 in MPC-D 107/15). Equivalent measurements (skull and jaw lengths) in MPC-D 100/42 (*Citipati* sp.) suggest that this virtually complete individual is also approximately the same size as the two *Nemegtomaia* specimens. The femur length in MPC-D 100/42 is 305 mm and its shaft circumference is 95 mm (which suggests the animal weighed 40 kg using the formulae of [41]), and the length of the skeleton is 1.95 meters. It is therefore likely that MPC-C 107/15 was an individual with a total length of about 2 meters, and that it weighed approximately 40 kg. The edges of the premaxilla, nasal, frontal and parietal clearly show that MPC-D 107/15 was a crested oviraptorid like *Citipati*, *Nemegtomaia* and *Rinchenia* [10], [17], [39]. The crest is similar to those of both *Citipati* and *Nemegtomaia* in that it is relatively low and is centered above the antorbital region, in contrast with the much taller crest of *Rinchenia* that is centered more posteriorly. Whereas crest development extends posteriorly to thicken and pneumatize the frontals in *Citipati* [40], this region is thin, flat and not pneumatized in MPC-D 107/15 and the holotype of *Nemegtomaia*. Like the holotype of *Nemegtomaia*, the ventral part of the anterior margin of the premaxilla of MPC-D 107/15 is nearly vertical to the main axis (as seen in lateral view) of the suborbital portion of the jugal. This is in contrast with known specimens of *Citipati*, where the anterior margin of the premaxilla slopes anterodorsally. The jugal process of the postorbital (Fig. 5a) forms the upper two thirds of the posterior border of the orbit in MPC-D 107/15 and most other oviraptorids, but *Citipati* is unusual in that the jugal process extends to the ventral margin of the orbit. As in *Nemegtomaia*, the ventral end of the exoccipital of MPC-D 107/15 (Fig. 5B) is level with the suborbital process of the jugal, which is considerably lower than in *Citipati*. Whereas *Citipati* is from Djadokhtan-aged strata of Dzamyn Khond and Ukhaa Tolgod, the holotype of *Nemegtomaia* came from the Nemegt Formation less than 500 m from where MPC-D 107/15 was collected. Similar morphology, similar size, geographic proximity, and the interfingering nature of the Baruungoyot and Nemegt formations make it most parsimonious to identify MPC-D 107/15 as *Nemegtomaia*.

Like all oviraptorids, the skull of MPC-D 107/15 is relatively short compared with other parts of the body (Figure 5). It is, for example, almost half the estimated length of the femur, whereas in other theropods the skull can be more than 90% the length of the femur. Oviraptorid skulls are short overall because of a significant reduction in the length of the antorbital region [10]. The eyes of oviraptorids look large because of the relatively small sizes of most species, but also because of their short skulls. If orbit length is plotted against femur length (logarithmically transforming the measurements) for 76 theropods (Currie, unpublished data), the resulting regression is defined by y = 0.6517x+0.0931 where y represents orbit length, x is femur length and 0.6517 is the allometric coefficient. The allometric coefficient shows that orbit length (roughly equivalent to the size of the eyeball) shows strong negative allometry with size increase. Adding the data for nine oviraptorosaurs where both these dimensions are known clearly demonstrates that the orbit length of each oviraptorid individual is not significantly different from what is expected in any theropod. The orbit of MPC-D 107/15 is 55 mm high, which suggests that the length was 50 mm (compared with 55 mm in the holotype). The anterior margins of the premaxillae are well preserved, and show that the external nares would have been relatively small and positioned high as in the holotype. The lateral temporal opening was clearly large and quadrangular (Figure 5). The antorbital length of MPC-D 107/15 is 78 mm, which is almost ten percent longer than in the holotype. A difference of this scale can be easily accounted for as individual variation or taphonomic distortion, and is not significant.

The anterior (premaxillary) and dorsal (nasal) margins of the skull show that MPC-D 107/15 had a prominent crest like many other oviraptorids, including the holotype of *Nemegtomaia* (MPC-D 100/2112), *Citipati* sp. (MPC-D 100/42) and *Rinchenia mongoliensis* (MPC-D 100/32-1). The specimen is not well enough preserved to determine whether or not the nasal was pneumatic, as it clearly was in *Rinchenia* and other mature oviraptorid individuals. The presence of a crest is generally associated with oviraptorines rather than ingeniines, but may in fact be correlated with size and maturity; most ingeniines are in fact smaller than most oviraptorines [2]. The anteroventral margin of the premaxilla is almost perpendicular to the ventral margin of the jugal (Figure 5). The height of the premaxilla below the external naris is 44.8 mm. The premaxillary-maxillary suture is nicely preserved on the right side of the skull (Figure 5). If a line is drawn through the ventral margins of the quadratojugal and maxilla (Fig. 5A), then the tomial margin of the premaxilla clearly projects well below the maxilla. This may ultimately turn out to be a size correlated character of little taxonomic utility. The lacrimal is platelike and projects lateral to the orbit in both MPC-D 107/15 and the holotype. Although the skull roof cannot be seen as well as in the holotype, the nasal of MPC-D 107/15 appears to have been no longer than the frontal. It appears unlikely that the frontals and parietals were pneumatized like the nasals, because the small sections of preserved surfaces of these bones are flat in MPC-D 107/15. There is no evidence for a separate prefrontal in either this specimen or the holotype. The postorbital process of the jugal extends posterodorsally from the suborbital process of the jugal (Figure 5). The basal tubera cannot be seen in MPC-D 107/15, but appear to be well developed in the holotype [14]. As in other oviraptorids, the jaw is deep (the maximum height of the dentary is a third of the jaw length), and the external mandibular fenestra is large, making up more than a quarter of the jaw length (Figure 5). A prominent process of the surangular invades the back margin of the external mandibular fenestra as in all oviraptorids.

**Figure 6:**
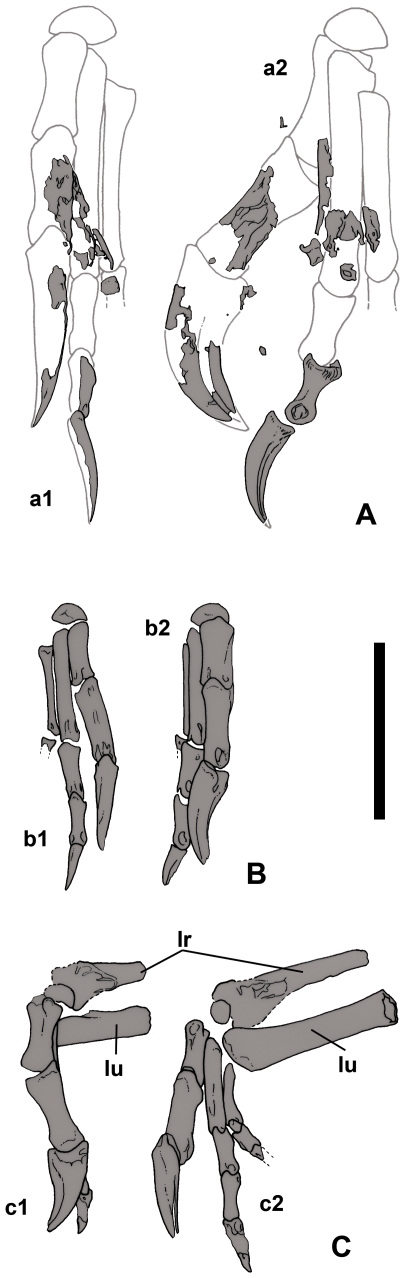
Hands of *Nemegtomaia barsboldi*. A, preserved elements and reconstructed manus of MPC-D 107/15 in dorsal (a1) and lateral (a2) views: B, right manus of MPC-D 107/16 in dorsal (b1) and medial (b2) views; C, left manus of MPC-D 107/16 in medial (c1) and dorsal (c2) views, with the preserved distal portions of radius and ulna. **lr**, left radius; **lu**, left ulna. Scale bar 5 cm. Illustration by Marco Auditore.

Postcranially, the ends of most bones were destroyed, presumably by insect scavengers (Figures 3, 4). Most of the scapula (180 mm) is present, and the total length appears to have been about 185 mm. The minimum shaft width is 15 mm, and the distal end expands to 27 mm. Although the full length of the humerus is not preserved, the minimum shaft width (transverse) has a diameter of 19 mm. Amongst sixteen oviraptorosaurs (Table S4), there is a strong correlation (r^2^ = 0.95) between humerus length and transverse shaft width, which suggests that the humerus would have been 152 mm long in MPC-D 107/15. The ratio of the lengths of the scapula to the humerus can therefore be calculated as 1.2, which is typical of the majority of oviraptorids. The distal end of the deltoid process is preserved, but little can be said other than it was well developed and extended far down the shaft as in other oviraptorids. Only small sections of the shafts of the radius (the diameter of the shaft width is 12 mm) and ulna (SW = 14 mm) are preserved, but show that the diameter of the shaft of the radius was 0.86 that of the ulna. The length of the radius was estimated as 144 mm using 10 pairs of measurements of oviraptorid radii, plus the measurements of radii from seven individuals of the oviraptorosaurs *Avimimus* and *Caudipteryx* (b = 1.35, k = 0.75, r^2^ = 0.89). The estimates based on shaft widths suggest that the ratio of radius length to humerus length is 0.95, which is close to the same ratios in *Citipati osmolskae* (MPC-D 100/979), and *Conchoraptor gracilis* (MPC-D 110/21). In contrast, the ratio is less than 0.80 in *Ingenia* (MPC-D 100/32, 100/33, 110/03) and *Rinchenia* (MPC-D 100/32-1). The ratio is intermediate between the two extremes in all other oviraptorid taxa. The hands were also badly damaged, but it is clear that the phalanges (I-1, I-2) of the first digit were more massive than those of the second digit (II-2, II-3) (Fig. 6A), that the distal end of the second digit does not extend far beyond the distal end of the first digit, and that the third metatarsal is slender. These are all characters that are diagnostic for Ingeniinae.

The pubic shaft is preserved in MPC-D 107/15, and was clearly curved anteroventrally (is anteriorly concave in lateral view) as in other oviraptorids.

Most of the femur is preserved, although the distal end is in poor condition (Fig. 4B). Its length can be estimated from the transverse diameter of the shaft (31 mm) and is approximately 286 mm when compared with 26 oviraptorids and dromaeosaurids (b = 1.22, k = 0.83, r^2^ = 0.95). The length of the femur is 10% longer (305 mm) in MPC-D 100/42 (Citipati sp.), which has similar dimensions in other parts of the skeleton to MPC-D 107/15. This suggests that the humerus was roughly half the length of the femur. The shaft width of the tibia, when compared with 23 other oviraptorids and dromaeosaurids (selected because they are not arctometatarsalian; b = 1.55, k = 0.64, r^2^ = 0.87), produces an estimated length of 317 mm for the tibia. This makes sense because it suggests that the tibia in the folded legs of MPC-D 107/15 (Fig. 4B) is about 5–10% longer than the femur as in other oviraptorids.

### Posture

MPC-D 107/15 shows no evidence of post-mortem transportation. It is preserved in a facies hypothesized to have been deposited in a single sandstorm or dune-shifting event. The body had shifted slightly to the right of center, rather than being exactly symmetrical across the midline of the skeleton; the right forearm and part of the right foot are obscured in dorsal view (Figure 3). This shift of the body suggests that the sediment deposited upon the skeleton may have come toward the animal from its left side. A similar assumption was made by Clark et al. [6] for another nesting oviraptorid (*Citipati osmolskae*, MPC-D 100/979) from Ukhaa Tolgod. Overall, MPC-D 107/15 shares several similarities with the brooding *Citipati* (MPC-D 100/1004) of Erickson et al. [8]. The neck curves toward the left and downward, and the skull is facing the outside of the nest in a position lower than much of its body (Fig. 3A,B). The humeri are oriented down and back, the radius and ulna of the left side are oriented forward and down (the right side is partially obscured by matrix), and the left hand is folded backwards. The forelimbs do not extend laterally as much as observed in other known nesting oviraptorid dinosaurs [4]–[6], [8]. The legs are folded up into a crouching position. The feet are upright and are medial to the position of preserved broken eggs. The tail, ischia, and presumably a large portion of the nest have been eroded away (Fig. 3C,D,E). In addition, the vertebral column and the neck, parts of the pelvic bones, ribs, and the vast majority of articular surfaces were scavenged before fossilization by saprophagus insects and/or other small scavengers such as small theropods or mammals. However, considering the overall preservation of the skeleton, the absence of all cervical, dorsal and sacral vertebrae is puzzling.

### Taphonomy

Specimen MPC-D 107/15 is a terrific source of information on taphonomic processes in the semi-arid environments of the Baruungoyot Formation. In fact, a combination of different factors resulted in the final preservation of the body: 1. lateral shift of the body due to mono-oriented sediment accumulation; 2. partial burial and subsequent deterioration (and possibly predation by scavengers) of vertebral column, neck, and hips; 3. gradual decomposition under xeric conditions; 4. intense scavenging by insect colonies and; 5. final burial. In addition to minor deformation caused by vertical compression during and after burial, much of the damage observed in the skeleton and nest were caused by intense activity of invertebrates. A number of studies have dealt with insect traces on fossil bone and their implication in constraining the reconstruction of the taphonomic history of a specimen [42]–[48]. Such studies also document bone modification during early stages of fossilization and indicate a common pattern in areas where damage is more extensive, and in particular in the cranial and dorsal vertebrae [49], [50]. Traces of dermestid beetles and possibly other saprophagus insect activity are documented by bone borings, bone-chip-lined burrows, and reworked sediment in specimen MPC-D 107/15 (Figure 4), some of which may have been caused by the construction of pupal chambers. Nearly circular borings passing completely through thin bones are preserved in the left side of the skull and range between 3 and 6 mm in diameter (Fig. 4D,E,F,G). Similar borings have been previously reported from the Baruungoyot Formation and coeval Djadokhta Formation [30], [49] on *Bagaceratops*, *Pinacosaurus*, *Protoceratops*, *Velociraptor*, and other ankylosaurid skeletons [6], [51]–[54]. Feeding traces are also abundant in the joints of the skeleton and nearly obliterate all articular surfaces (Fig. 4A,B,C). Bone-chip-lined burrows have been found around the skeleton, and the largest of these are approximately 35 mm in diameter and contain fragments of bone up to 7 mm wide (Fig. 4G). Furthermore, a large section of the nest revealed reworked sediment in the form of a light colored, elongate tunnel under the neck and the skull: interestingly, such larger traces are found only in the part of the nest where neither eggs nor egg fragments have been recovered (Fig. 4H). Similar to several specimens collected in the Gobi desert of Mongolia and China [48], [55], no pupal chambers or similar structures have been observed. As pointed out by Hasiotis et al. [44], dermestid beetle activity implies specific environmental conditions. Present day dermestids, for instance, feed primarily on dried muscle tissue and they do not eat moist materials; furthermore, rapid burial prevents any activity [56]. The presence of such intense damage to bones supports the hypothesis that the carcass was partially buried with the upper part of the body exposed and subject to rapid exsiccation and deterioration for long enough to allow a dermestid colony to develop. If skeletons are kept in dermestid colonies for long enough, depending on environmental and seasonal conditions, articular surfaces and thinner bones (such as skull, ribs, scapulae, etc.) are totally destroyed by the beetles [44], [57]. Recently, Saneyoshi et al. [49] suggested that some of the largest borings found on a *Protoceratops* skeleton collected from the aeolian beds of the Djadokhta Formation at Tugrikin Shire can be referred to scavenging activities of small mammals. Similarly, remains of small mammals have been found in the Baruungoyot and Nemegt formations of the Trans-Altai Gobi [35], [58]. Thus, it is possible to consider small mammals as responsible for bone damage observed in MPC-D 107/15, and in particular for the damage observed in the vertebral column.

### Description and taphonomy of specimen MPC-D 107/16

**Figure 7:**
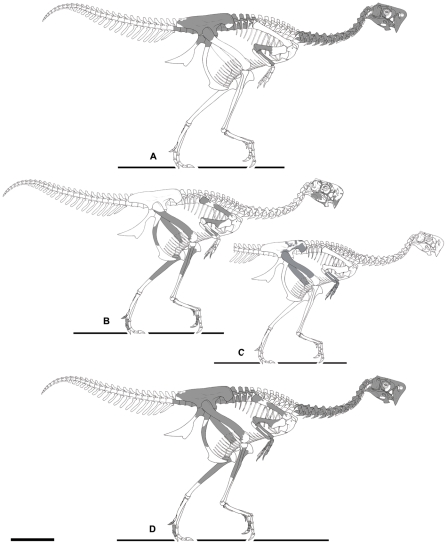
Skeletons of *Nemegtomaia barsboldi* specimens (known elements in grey). A, the holotype (MPC-D 100/2112); B, MPC-D 107/15 (the left side of the skull, left forelimb, pubis and toes are reversed and superimposed to the right side). C, MPC-D 107/16 (the left forelimb is reversed and superimposed to the right side). In D, the preserved elements of MPC-D 107/15 and 16 are scaled to and superimposed on the holotype (the skull depicted is that of the better preserved MPC-D 100/2112). Scale bar 20 cm. Illustration by Marco Auditore.

MPC-D 107/16 is a partial skeleton of another oviraptorid from the Baruungoyot Formation. Most of the skeleton had eroded away before it was discovered, but half a dozen ribs, the anterior edge of the right ilium, the distal ends of the left radius and ulna, both hands, and most of both femora (Figs. 6, 7) were recovered. The specimen can be identified as *Nemegtomaia* on the basis of its hands, which have all the same ingeniine characteristics as MPC-D 107/15. The diagnostic features of the hand that these two specimens share include the relatively large first digit with a strong ungual, and a third digit that is so small that the third metacarpal is reduced to a splint. The specimen is significant in that it provides information on the anatomy of *Nemegtomaia* that is not available with either the holotype or MPC-D 107/15. It is also approximately 35% smaller than either of the other two specimens.

The rib fragments all appear to be from the right side, and the spacing suggests that they were buried during the Cretaceous as part of a fully articulated skeleton. The positions of the anteroventral edge of the right ilium, and the right femur also suggest that this is true. However, in the same block of sandstone is the articulated right hand, but it is far enough behind the right leg to suggest that either it has been separated from the specimen, or that it represents a second individual of the same size. The orientation of the forearm and hand suggests the latter may be true. The articulated left hand was found in another block of sandstone, and therefore does not help to establish whether there are one or two individuals. Both hands are the same size, and are from an individual that was the same approximate size as the hind limbs. The most parsimonious interpretation would be that all elements represent a single individual because dinosaur skeletons recovered from the Baruungoyot Formation tend to be articulated but are not normally associated with each other. However, multiple oviraptorid skeletons have been found in single quarries in nearby parts of Mongolia (including a pair of *Ingenia* collected at Khermeen Tsav [MPC-D 102/02, MPC-D 102/03] by an earlier “Dinosaurs of the Gobi” expedition, and a pair of *Khaan* excavated by the American Museum of Natural History at Ukaa Tolgod [39]. For the purposes of this paper, we are assuming that MPC-D 107/16 represents a single, somewhat disarticulated individual.

The shafts and distal ends of the left ulna and radius are preserved in articulation with the hand, which is folded perpendicular to the forelimb (Fig. 6C). The ulna has a straight shaft like those of *Heyuannia* and *Ingenia* but is distinctive from the bowed shafts found in most oviraptorids. The diameter of the shaft of the radius is 78% that of the ulna. Using the same formula that was used to estimate the length of the radius of MPC-D 107/15, we can estimate the length of the radius as 99 mm in MPC-D 107/16.

The length of the hand (mc II+phalanges II-1, II-2, II-3) of *Nemegtomaia* (MPC-D 107/16) is relatively short for an oviraptorid, and is 36% the length of the femur, which is comparable with *Ingenia yanshini* (40% in MPC-D 100/33). This suggests that the hand overall is shorter than in *Khaan mckennai* (MPC-D 100/1002, 74%) and *Citipati n.sp.* (MPC-D 100/42, 97%).

MPC-D 107/16 shows that *Nemegtomaia* had at least three carpal bones. The semilunate carpal of the right hand is still in position capping the first and second metacarpals (Fig. 6B,C). Like *Machairasaurus* [11], the semilunate carpal protrudes between the proximal ends of metacarpals I and II, and the distal articular facets are angled away from each other. Three carpals are visible in the left hand. One is presumably the semilunate carpal but it is partially obscured by matrix. It has drifted a few millimeters away from the metacarpus, and therefore was clearly not fused to it. The semilunate carpal often fuses with the first two metacarpals in oviraptorids, including specimens of *Heyuannia* [19] and *Ingenia* (MPC-D 100/30). This is clearly an age related phenomenon, and fusion may have occurred in larger specimens of *Nemegtomaia*. The second carpal is a small, round bone positioned between the distal ends of the ulna and radius. The identity of the third carpal is uncertain because it is small, round and featureless, and has drifted out of position to lie on the proximodorsal surface of the first metacarpal.

The metacarpus is well preserved in both hands. The first metacarpal is slightly more than half the length (52%) of the second metacarpal, which is comparable with *Conchoraptor* (58% in MPC-D 100/38), *Heyuannia* [19], *Ingenia* (69% in MPC-D 100/30), and *Machairasaurus* (63% in IVPP 15979). The percentage is less than fifty in other oviraptorids. Even though it is shorter than the other metacarpals, the shaft of metacarpal I is 50% thicker than that of metacarpal II, and is four times thicker than the shaft of metacarpal III. The same shaft thickness ratios are slightly less in *Ingenia* (1.38 for McII and 2.93 for McIII in MPC-D 100/33) and *Heyuannia* [19]. The proximal end of the first metacarpal is divided into a large proximolaterally oriented facet for contact with the semilunate carpal and a smaller proximomedially facing surface.

Metacarpal III is about 90% the length of McII. However, its proximal end is more distal in position than that of McII; consequently, the distal end of McIII almost reaches the distal end of McII. The proximal end of metacarpal III is also separated from the carpus in *Heyuannia* [19], *Khaan* [39], and *Machairasaurus* [11]. The shafts of metacarpals II and III are closely appressed as in other oviraptorids. As in MPC-D 107/15, the shaft of the third metacarpal is reduced to a splint.

The first digit is almost as long as the second digit, with the tip of the first ungual reaching mid-length the second ungual, as it does in the articulated hand of MPC-D 107/15. In comparison, *Citipati*, *Khaan* and *Machairasaurus* each have a first ungual that extends to the end of the penultimate phalanx of digit II, whereas in *Ingenia* the tip of the first ungual reaches the same level as the tip of the second one. The first phalanx of the first manual digit is robust, and is about 40% thicker than the first phalanx of the second digit. In *Heyuannia* and *Ingenia*, manual phalanx I-1 is double the diameter of phalanx II-1. In the second digit of MPC-D 107/15, the combined lengths of phalanges II-1 and II-2 equal 29.5 mm, which is shorter than the length (34 mm) of the second metacarpal (87%). This is also true for *Ingenia* (MPC-D 100/30, 78%; MPC-D 100/31, 76%; MPC-D 100/33, 77%) and *Heyuannia* [figure 13.4 in 19], in which the sum of the lengths of these two phalanges is about the same as the length of the associated second metacarpal; in all other oviraptorosaurids, the sum is greater. Phalanx II-2 is shorter than phalanx II-1, which is similar to the situations in *Heyuannia*, *Ingenia* and *Machairasaurus*; in most of the other oviraptorids, phalanx II-2 is longer than phalanx II-1. The third digit of the right hand, except for the base of the first phalanx, was destroyed by erosion. There are at least two small phalanges on the third digit of the left hand, but the distal end of the second one is incomplete. In the left hand of MPC-D 107/15, and both hands of MPC-D 107/16, III-1 is less than two thirds the length of II-1 and almost half its diameter.

Only the anteroventral corner of the preacetabular process of the ilium is preserved, but it is similar to the shape of those parts of the ilia of the holotypes of *Nemegtomaia* and *Ingenia*.

The femora are almost complete and as preserved are up to 21 cm long. Using the transverse shaft width and the same regression that was used for MPC-D 107/15, the calculated length of the femur is 222 mm.

### Phylogenetic analysis

**Figure 8:**
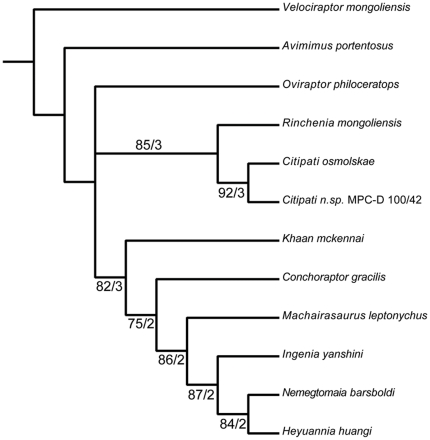
Phylogeny of Oviraptoridae. Phylogenetic analysis of Oviraptoridae was performed by adapting the matrix of Longrich et al. (3) and using the exhaustive search algorithm of PAUP* 4.0b10. Numbers next to clades indicate bootstrap support value/decay value.

Phylogenetic analysis was performed by adapting the matrix of Longrich et al. [11] and using the exhaustive search algorithm of PAUP* 4.0b10 [59] (Figure 8) (Tables S1, S2, and S3). *Velociraptor* was used as the outgroup for the Oviraptorosauria, *Avimimus* was used as the outgroup for the Oviraptoridae, and taxa (*Chirostenotes*, *Gigantoraptor*, *Incisivosaurus* and *Nomingia*) were excluded if fewer than 50% of the 183 characters (Table S2) were coded. *Heyuannia*, *Machairasaurus* and *Oviraptor*, also had fewer than 50% of the characters coded, but were left in because they are part of the ingroup being analyzed. The subset included twelve taxa (*Avimimus portentosus*, *Citipati osmolskae*, *Citipati* n.sp. (MPC-D 100/42, also referred to in other analyses as “*Oviraptor philoceratops*” or the Zamyn Khondt [ = Dzamyn Khond] oviraptorid), *Conchoraptor gracilis*, *Heyuannia huangi*, *Ingenia yanshini*, *Khaan mckennai*, *Machairasaurus leptonychus*, *Nemegtomaia barsboldi*, *Oviraptor philoceratops*, *Rinchenia mongoliensis* and *Velociraptor mongoliensis*). Three characters are ordered, and the remaining ones are unordered. All characters have equal weight. A total of 654,729,075 trees were evaluated to produce two most parsimonious trees of length 210 (consistency index of 0.867, a retention index of 0.731 and a rescaled consistency index of 0.633). The only difference between the two trees is that *Oviraptor* clustered in one tree with *Rinchenia mongoliensis*, *Citipati osmolskae* and *Citipati* n.sp. in a monophyletic Oviraptorinae, whereas in the other tree *Oviraptor* was the sister to all other oviraptorids. The Ingeniinae includes (*Khaan*+(*Conchoraptor*+(*Machairasaurus*+(*Ingenia*+(*Heyuannia*+*Nemegtomaia*))))).

Robustness was determined using bootstrap (1000 replicates) and decay indices (Fig. 8). All ingroup nodes have decay values greater than 1, and are still present in the strict consensus of all trees either one or two steps beyond the Most Parsimonious Tree. With the exception of *Oviraptor*, all bootstrap values within the Oviraptoridae have high bootstrap values (greater than 75%).

In all runs of the phylogenetic analysis, *Nemegtomaia* is shown to be one of the most derived ingeniine oviraptorids, and is most closely related to *Ingenia* and *Heyuannia*. The original analysis by Lu et al. [14] recovered *Nemegtomaia* as an oviraptorine oviraptorid closely related to *Citipati osmolskae*, thereby demonstrating that the additional anatomical information from the new specimens significantly improves our understanding of oviraptorid systematics (Figure 8).

Some of the characters in the Longrich et al. [11] matrix rely on ratios of bones that are incomplete in MPC-D 107/15, and to code the characters, missing dimensions were calculated from the parts of the bones that are preserved. Five of these characters (130, 134, 136, 138, 139) were coded, and may have unduly influenced the results. Because this is a potential source of error, the five characters were rescored as unknown for *Nemegtomaia*, and the analysis was rerun. This time the phylogenetic analysis was performed using the heuristic search algorithm of PAUP* 4.0b10 [59] using 1000 replicates, and once again the analysis produced two most parsimonious trees with tree lengths of 210. The consensus tree topology is identical to that of the previous analysis, the CI remained the same at 0.867, and the RI is slightly less at 0.728. With the exception of *Oviraptor*, all bootstrap values (1000 replicates) within the Oviraptoridae still have high bootstrap values, although each dropped in value by 2 to 4%.

### The Nest

**Figure 9:**
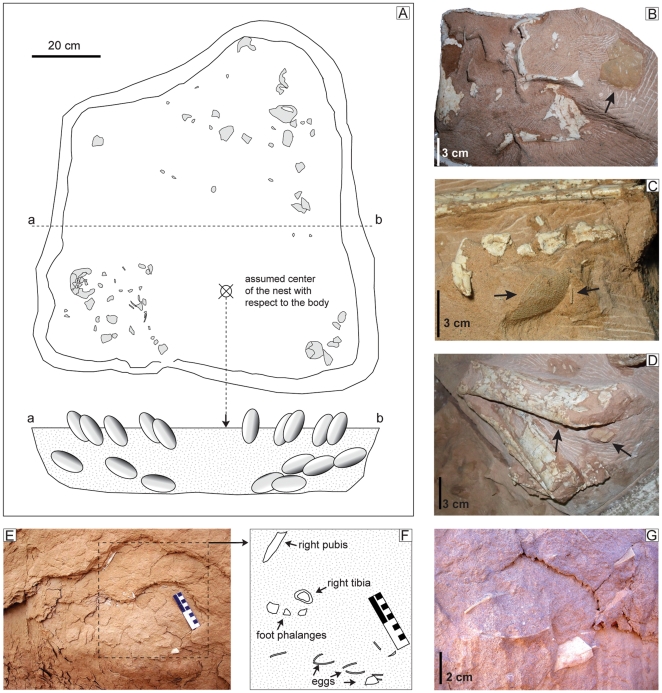
The nest of *Nemegtomaia barsboldi*, MPC-D 107/15. A, disposition of preserved eggs within the nest. Eggshells have been recovered under the skull (B), left pes (C) and leg (D), suggesting the direct apposition of the oviraptorid on top of the eggs. During the early excavation of the nest, it was possible to document a lower layer of eggs lying approximately 10 cm below the body (E–G).

The collected portion of the nest is approximately 90 cm wide and 100 cm long (Figs. 3, 9A). If we consider as paleo-ground level the virtual plane where feet and head lie, the skeleton occupies the upper 25 cm, whereas the remaining 20 centimeters of the block are entirely occupied by broken eggs and eggshells. Within the nest there is no obvious variation in sedimentary structures nor changes in micro- and macroscopic details. There is also no evidence of plant material, whereas several undetermined bone fragments were recovered within the sediment in the nest and immediately above the skeleton. Similar to several nests collected from the Gobi desert of China and Mongolia [4]–[8], [60], [61], specimen MPC-D 107/15 does not preserve a single complete egg, nor have any embryonic skeletal elements been recovered [20], [21]. Such poor preservation prevents one from estimating the size and shape of a single egg; determining the number of eggs laid, and observing specific orientations or patterns in the arrangement of the eggs within the nest. Overall thickness of the block, and field observations of the specimen suggest that two layers of eggs were originally preserved below the body. Most of the center of the nest is not exposed; nevertheless, there is no evidence of eggs in the center at the same level as the exposed eggs. The plaster and burlap jacket protecting the specimen precludes direct observation of parts of the nest that were observable in the field. A total of seven distinct eggs were identified in the lower layer where damage by dermestid activity is assumed to be minor (Fig. 9E, F, G). Large fragments of eggs were recovered under the skull, left side of the neck, left humerus, left femur, and both feet: in all cases, the bones rest directly on or within 5 mm from the surfaces of the eggs (Figure 9). The direct apposition of the skeleton on the nest in MPC-D 107/15 shows that the nest was not completely covered by sand. It is important to note that the majority of egg fragments are located in three distinct sections of the nest—beside the left and right legs, and in front of the shoulder girdle and neck. In the majority of known oviraptorid nests, eggs are arranged in pairs at different levels in up to three concentric circles [60], [62]. This is not the case of MPC-D 107/15, where the positions of the eggs do not suggest a specific arrangement. Eggs were likely displaced during early stages of burial by external factors (such as strong winds, sediment transport associated with sandstorm events or small predators). This supports the conclusion that the upper layer of eggs was not buried (or was only partially buried) because it is unlikely that endogenous factors were able to interfere with fully buried eggs. Previous studies [4], [9], [23] proposed the hypothesis that several individuals gathered eggs into a single nest and arranged them so they could be protected by one individual, possibly a male. Although it is possible that *Nemegtomaia* laid eggs with no particular arrangement within the nest, this seems unlikely given the large numbers of oviraptorid nests that have been found in Cretaceous deposits of China and Mongolia. Specimen MPC-D 107/15 is a *Nemegtomaia* individual associated with a nest of eggs and therefore *Nemegtomaia* represents the fourth known genus of oviraptorids (*Citipati*, 8; cf. *Machairasaurus*, 5; *Oviraptor*, 1; *Nemegtomaia*, this paper), the first within the Ingeniinae clade, found on nests.

### Eggs

**Figure 10:**
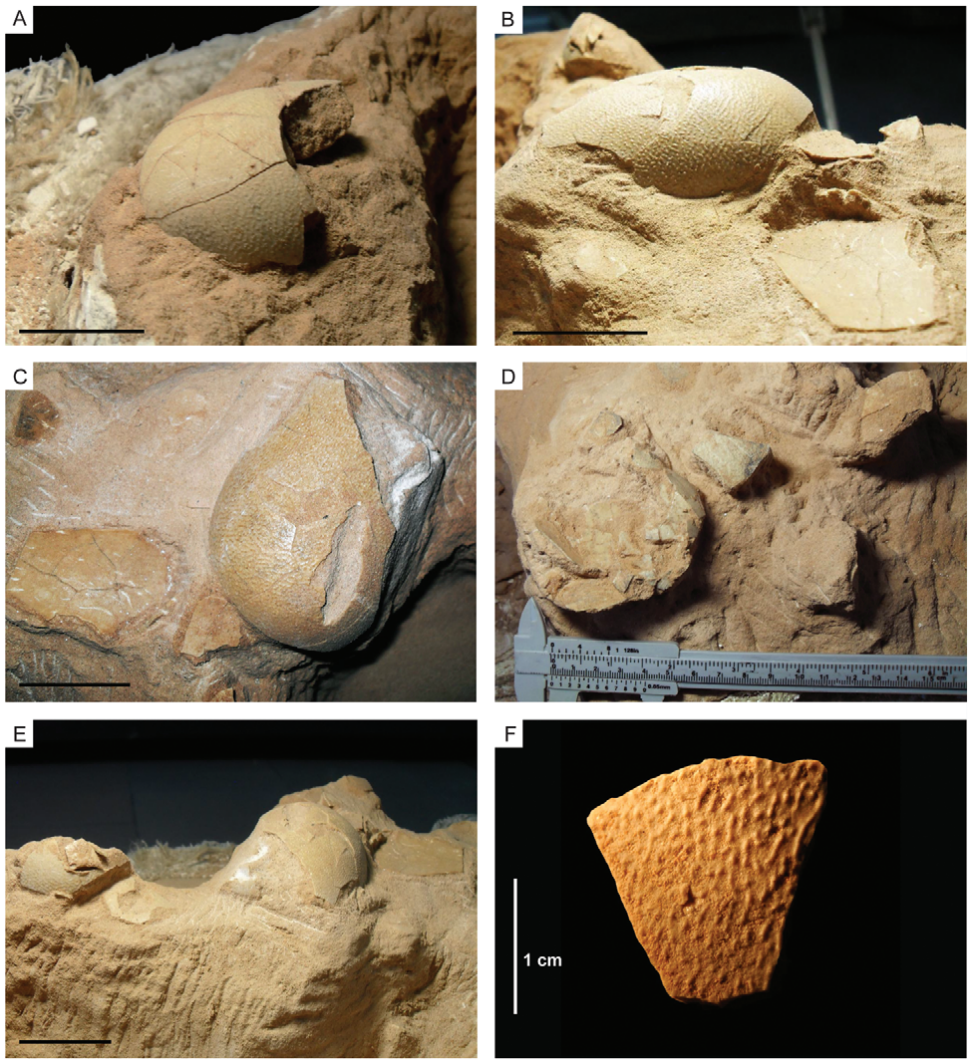
Eggs of *Nemegtomaia barsboldi*. Details of eggs preserved in the upper layer of eggs in specimen MPC-D 107/15. Scale bar A–E 5 cm.

None of the eggs is preserved in its entirety, and therefore all of the dimensions of an egg cannot be measured accurately (Fig. 9A). Available data on oviraptorid eggs in the literature [5]–[7] [20] [21] [60] [63] [64] indicate an average length of 17 cm (min = 11.5 cm, max = 25 cm), and a width of 7 cm (min = 5 cm, max = 8.5 cm). By comparison, the most complete eggs found in specimen MPC-D 107/15 are estimate to be 5 to 6 cm wide and 14 to 16 cm long. Preserved eggshells are macroscopically nearly identical to those described by Norell et al. [20], [21] and can be referred to the elongatoolithid oofamily [60], [65], [66], originally considered to be the ornithoid basic type (Figure 10). Eggshells are relatively thin, ranging between 1 and 1.2 mm. The outer surfaces of the eggs are ornamented with linearituberculate ridges and nodes that rise approximately 0.3 mm above the shell (Fig. 10F). Such longitudinal ornamentations do not show any specific trend or variation from the equatorial to apical region. Eggshell microstructure from a total of eight fragments collected from the nest were studied in tangential and radial thin sections, using optical and polarizing light microscopy, and scanning electron microscopy. Unfortunately, all analyzed fragments were heavily altered and re-crystallized calcite, and all histostructures, including pore canals, were obliterated. Furthermore, no pores were observed on the external surfaces of the eggshells.

## Discussion

The Nemegt locality provides excellent exposures of the transitional interval between the Baruungoyot and Nemegt formations, and in particular the gradual southeastward progradation of the Nemegt Formation depositional system (characterized by channel-fill and sheet-flood deposits) on top of the Baruungoyot Formation (dominated by semi-arid or arid environments with recurrent aeolian beds) [33]. This interval is also the most fossil-rich vertebrate stratigraphic section in the area, hosting most of the vertebrates observed in the Nemegt beds [33]. This assumption draws attention to the palaeobiology of faunal assemblages recovered from this interval. In particular, oviraptorids of Mongolia and China have been collected primarily from deposits representing semi-arid or xeric environments [1], [4]–[7], [10]–[12], [20], [39], [67]–[70]. The sole exception was represented by the type specimen of *Nemegtomaia*, which was collected from the fluvially-dominated Nemegt Formation. Insufficient data are available at present for the type specimen of *Heyuannia huangi* [68], [71], recovered from the? Maastrichtian Zhutian Formation in southern China. However, this study suggests that *Nemegtomaia* is found in the typical upper Baruungoyot Formation facies, in the Baruungoyot-Nemegt interfingering interval, and also in the Nemegt Formation. Therefore, this study provides additional evidence that both environments co-existed laterally, and that the generally distinctive faunas from the two units do overlap as in the case of *Avimimus*, *Ingenia*, *Nemegtomaia*, and *Tarchia* [30], [33], [36], [72], [73]. The occurrence of a *Nemegtomaia* nest in this stratigraphic interval may indicate that oviraptorid nesting grounds were chosen near permanent or seasonal streams that provided soft and sandy substrate as well as a source of food within an otherwise xeric environment. The large number of adult oviraptorids found in brooding positions may indicate that they brooded their clutches for relatively long periods of time. This is similar to some present day birds from arid regions of Africa and Australia (such as the ostrich [*Struthio camelus*], the emu [*Dromaius novahollandiae*], and the black-breasted buzzard [*Hamirostra melanosternon*]), which incubate their brood for more than 40 days with limited food and water supplies. In desert environments, thermal stress may be most critical during reproduction, as the adult is restricted to the nest for large portions of the day. Furthermore, eggs and nestlings are particularly susceptible to thermal damage. Mechanisms allowing successful incubation in extreme heat, such as a specific choice of the nesting area, must have existed in the Late Cretaceous Gobi. One suggestion for the appearance and evolution of rectrices in oviraptorosaurs is as an adaptation to shade and protect eggs in the nest [74], [75].

The new specimens demonstrate that *Nemegtomaia* is similar to derived ingeniine oviraptorids like *Heyuannia*, *Ingenia* and *Machairasaurus* in having a robust, relatively short manus with a robust metacarpal I that had a powerful ungual. There are relatively few oviraptorid specimens (eight) that have both femora and hands preserved. Nevertheless, regression analysis of a suite of theropods shows that the lengths of the second metacarpals in *Citipati* and *Khaan* are close to what would be expected in a velociraptorine dromaeosaurid, whereas the lengths of the metacarpi are reduced in *Ingenia* and *Nemegtomaia*. The relative lengths of different elements of the hand of ingeniines should be thought of in terms of the relative shortening of the second digit rather than the lengthening of the third metacarpal. During theropod evolution, the first metacarpal becomes relatively shorter in relation to the length of the second metacarpal. An ingeniine seems to have a relatively long first metacarpal compared with the second metacarpal, but this is not a reversal in the theropod trend because it is accomplished in a different manner (through the shortening of the second metacarpal rather than by increasing the length of the first metacarpal). Nevertheless, there are changes in the ingeniine metacarpal I in that it becomes relatively thicker to support a more robust finger, and it expands to cover the posteroventral surface of the second metacarpal at the proximal end. This presumably immobilizes the metacarpus, and in the holotype of *Ingenia* (MPC-D 100/30), this is taken a step further through coossification of the semilunate carpal with the three metacarpals. The phalanges in digit II also become relatively shorter in comparison with the second metacarpal so that the finger overall is also shortened. This presumably is associated with a change in function, and may have something to do with the presence of long remiges behind the forelimb that also attach to the second manual digit. A similar trend of digital reduction (especially in the ungual) can be found in *Confuciusornis* [76]. As the second digit assumed the function of feather support, it reduced its capability for using it as a grasping appendage. This function seems to have been largely taken over by the first digit, which is probably why it became more robust in the Ingeniinae. The size reduction of the third finger is a logical consequence of the feathered hand because it would be positioned above and behind the feathers where it could not be used effectively for grasping.

*Nemegtomaia barsboldi* is the only member of the subfamily Ingeniinae that is known to have a crest. Crestless skulls of other ingeniines (*Conchoraptor*, *Heyuannia* and *Khaan*) are smaller than either of the two skulls of *Nemegtomaia*, which makes it possible that crest development is controlled by ontogeny or by absolute size. The crests tend to be highly pneumatic, and the presence of pneumatic nasals and frontals in at least *Conchoraptor* suggests that they had the potential for inflating these bones into crests in larger, more mature individuals. This taxon is known from the Baruungoyot Formation, from which at least two specimens of *Nemegtomaia* have been recovered. However, many skulls and skeletons of *Conchoraptor* have been collected in recent years and they are all of the same uniform small size. Their numbers, and the fact that at least one of the *Ingenia* specimens (MPC-D 100/30) has a coossified carpometacarpus, suggest that these animals probably never became as large as *Nemegtomaia*.

Ingeniinae are characterized by having seven or eight vertebrae incorporated into the synsacrum, whereas other oviraptorids have only six vertebrae incorporated into the sacrum. Irrespective of size, ingeniines have longer deltopectoral crests, more bowed ulnar shafts, and shorter hands with more powerful first manual digits.

### Conclusions

*Nemegtomaia* is the fourth genus of an oviraptorid dinosaur that has been found on top of a nest, but is the first within the Ingeniinae clade. Although the name means “good mother of Nemegt”, it received that name before there was any association between adults and eggs. Although the phylogenetic analysis of Lu et al. [19] found that *Nemegtomaia* was most closely related to the oviraptorine *Citipati*, the recovery of additional specimens clearly shows it to be an ingeniine oviraptorid. The hands of Ingeniinae genera are shorter than those of oviraptorines, but have more robust first digits. The more powerful claw on the first manual digit, the reduction in the length of the second manual digit, and reduction in size of the third finger are probably characters correlated with the presence of rectrices on the forelimb of these oviraptorids that were possibly used to protect the eggs in nests. Oviraptorid dinosaurs were one of the most common types of animals in arid environments during Late Cretaceous times of central Asia; nesting areas may have been selected with respect to nearby channel systems.

## Supporting Information

Table S1 **Characters modified from, or added to, the character list of Longrich et al.** [**11**] **.** Additional data on for caenagnathids as in [77] and for *Heyuannia* as in [78].
(DOC)

Table S2 **Character description.** Characters for phylogenetic analysis of the relationships among oviraptorosauria (modified after Longrich et al. [11]).
(DOC)

Table S3 **Character-taxon matrix.**
(DOC)

Table S4 **Selected measurements of oviraptorids.** Selected measurements of oviraptorids used for calculating the lengths of missing elements in MPC-D 107/15 and MPC-D 107/16. Measures in millimeters (PJC). Abbreviations: **SL**, skull length (preferably from the edge of paraoccipital process to the tip of the premaxilla, but in most cases taken from the quadrate to the premaxilla); **Pm1**, premaxilla height (below naris); **Pm2**, premaxilla height; **Ao sl**, antorbital skull length; **o l**, orbital length; **d**, dentary length; **d 2**, dentary maximum height; **j l**, jaw length; **sc l**, scapula blade length; **sc sw**, scapula shaft width; **h l**, humerus length; **h sw**, humerus shaft width; **r l**, radius length; **r sw**, radius shaft width; **u l**, ulna length; **u sw**, ulna shaft width; **mc I**, metacarpal 1; **mc II**, metacarpal 2; **mc III**, metacarpal 3; **I-1**, first digit, first phalanx; **I-2**, first digit, second phalanx; **II-1**, second digit, first phalanx; **II-2**, second digit, second phalanx; **II-3**, second digit, third phalanx; **III-1**, third digit, first phalanx; **il l**, ilium length; **f l**, femur length; **f sw**, femur shaft width; **f c**, femur shaft circumference; **t l**, tibia length; **t sw**, tibia shaft width; **III-3**, digit three, third phalanx; **IV-2**, digit four, second phalanx; **IV-3**, digit four, third phalanx; **IV-4**, digit four, fourth phalanx; **IV-5**, digit four, fifth phalanx. Additional data on oviraptorid specimens: *Gigantoraptor erlianensis* [79]; *Hagryphus giganteus* [80]; *Heyuannia huangi* [81]; *Oviraptor philoceratops* [1]; *Oviraptor* incertae sedis [82].
(XLS)
