# Supplementary material for: New Specimens of Nemegtomaia from the Baruungoyot and Nemegt Formations (Late Cretaceous) of Mongolia
Source: PLoS One. 2012 Feb 8;7(2):e31330. doi: 10.1371/journal.pone.0031330 (PMC3275628; doi:10.1371/journal.pone.0031330)
Supplement: Table S1 — Characters modified from, or added to, the character list of Longrich et al. [11] . Additional data on for caenagnathids as in [77] and for Heyuannia as in [78]. (DOC) [file pone.0031330.s001.doc]

**Table S1**

**Characters modified from, or added to, the character list of Longrich et al. [11].**

Character 1. *Ingenia*, based on numerous specimens, has a skull whose preorbital length is less than a third the basal skull length. This taxon is coded as “1”.

Character 2. *Ingenia*, based on numerous specimens, lacks a pneumatized cranial crest. This taxon is coded as “0”.

Character 4. The ratio of premaxillary tomial margin length to premaxillary height (below the external naris) is 0.5 in *Ingenia* (BHI specimen) and should be coded as “2”.

Character 5. The anterior margin of the premaxilla is vertical to the ventral margin of the jugal in *Ingenia* (BHI specimen) and should be coded as “0”.

Character 9. The length of the maxilla compared to skull length is less than 0.4 in both *Oviraptor* [40] and *Ingenia* (BHI specimen) and should be coded as “1” in both.

Character 16 has been rewritten as “Nasal along midline: longer than frontal (0); shorter than or as long as total length of frontal (1).” This seems to be true for all oviraptorids including *Citipati* [40] and *Ingenia* (BHI specimen), but possibly with the exception of *Nemegtomaia* (19), although the skull roof of the latter is poorly preserved.

Character 19. The narial opening of *Ingenia* (BHI specimen) is longer than high.

Character 41. The angle between the ascending and jugal processes of the quadratojugal is less than 90 degrees in *Ingenia* (BHI specimen, MPC-D PJC2002.17).

Character 53. The basioccipital processes are well developed and widely separated in Citipati [40].

Character 74. Most theropods have a vertical or slightly inclined mandibular symphysis. In oviraptorids, the U-shaped symphysis is broad and inclined to form a scoop-like process that is referred to in this paper as a “rudimentary symphysial shelf”. In caenagnathids, the connection is extended posteroventrally by a distinct horizontal shelf the spans the gap between mandibles [77]. All oviraptorids plus *Avimimus* are coded as “1”, and caenagnathids as “2”.

Character 81. “Process of surangular dividing external mandibular fenestra: absent (0); long (1); short (2) (Ordered)” needs to be rewritten as “Process of surangular dividing external mandibular fenestra: absent (0); short (1); long (2) (Ordered)” but the coding will remain the same. *Ingenia* (BHI specimen, MPC-D PJC2002.17) should be coded as “1”.

Character 83. Unless the jaws are crushed, all oviraptorids had mandibles that bow laterally at mid-length. This character has been re-coded as “1” for *Citipati*, *Conchoraptor*, *Ingenia*, *Khaan* and other taxa with mandibles preserved well enough to show this feature.

Character 95. Because the anterior extent of the mandibular adductor fossa is determined by the length of the surangular and the size of the external adductor fenestra, it is clear that this character should be coded as “1” for *Caudipteryx*.

Character 104. The cervical ribs of *Nemegtomaia* are firmly fused to the cervical vertebrae [78] and are coded as “2”.

Character 126. In HYMV1-2 (*Heyuannia*), the ratio of radius over humerus is 0.846, which should be coded as “1” rather than “0”.

Character 131. In *Heyuannia*, the second metacarpal is about the same length as the forearm [19, Fig. 13.3], and the forearm is 85% the length of the humerus. This suggests that the ratio of McII to the humerus in this genus should be coded as “1”.

Character 134. In HYMV1-2 (*Heyuannia*), the ratio of humerus to femur is 0.51, so Character 134 should be coded as “0”.

Character 138. In the *Ingenia* specimen with the number MPC-D 100/32 (note that this is a different specimen than the holotype of *Rinchenia mongoliensis*, which has the same number), a line drawn between the ventral margin of the postacetabular process of the ilium and the dorsal margin of the acetabulum intersects the anteroventral tip of the preacetabular process. This character has therefore been recoded as “0”.

Character 141. The posterior margins of both ilia of the holotype of *Nemegtomaia* (MPC-D 100/2112) are incomplete, but the contours of the postacetabular process suggest that it was “truncated or broadly rounded” rather than “acuminate”. The character has been coded as “0”.

Character 170. “External mandibular fenestra elongate (0) or height of external mandibular fenestra subequal to length (1).” Subequal is considered to be when the height falls within plus or minus 10% of the length, whereas in the case of *Conchoraptor gracilis* (ZPAL MgD-I/95) the length is more than 30% the height, which is therefore coded as “0”.

Character 174. Shaft thickness ratios for mtc I versus Mtc II in *Nemegtomaia* (1.5), *Ingenia* (1.4 in MPC-D 100/33) and *Heyuannia* [78] are robust, but not at the level of 200%. The character is rewritten as “Manus I-1 slender (0) or more robust (100 to 139 per cent) than II (1) or more than 140 per cent diameter of II-1 (2) ORDERED.

Character 177 has been changed to only two character states, with state “0” representing the situation where the sum of the combined lengths of manual phalanges II-1 and II-2 is subequal or more than the length of the second metacarpal, whereas the sum in state “1” is less than the length of the second metacarpal.
