# Supplementary material for: New Specimens of Nemegtomaia from the Baruungoyot and Nemegt Formations (Late Cretaceous) of Mongolia
Source: PLoS One. 2012 Feb 8;7(2):e31330. doi: 10.1371/journal.pone.0031330 (PMC3275628; doi:10.1371/journal.pone.0031330)
Supplement: Table S2 — Character description. Characters for phylogenetic analysis of the relationships among oviraptorosauria (modified after Longrich et al. [11]). (DOC) [file pone.0031330.s002.doc]

**Table S2**

**Character description**

1. Ratio of preorbital skull length to basal skull length: 0.60 or more (0); 0.59 or less (1). Note that an extra decimal point has been added to the figures of Osmólska et al. [10] for greater specificity. In the holotype of *Nemegtomaia*, the ration is 0.47.

2. Pneumatized crestlike prominence on the skull roof: absent (0); present (1).

3. Ratio of width (across premaxilla-maxilla suture) of snout to length: 0.3 or less (0); 0.31–0.49 (1); 0.5 or more (2). Character 3 is listed in Osmólska et al. [10] as 0.3-0.4 for character state 1, and 0.5 or more for character state 2 and is changed here to eliminate potential overlap.

4. Ratio of length of tomial margin of premaxilla to premaxillary height (below external naris): 1.0–1.4 (0); more than 1.7 (1); 0.7 or less (2). In the holotype of *Nemegtomaia*, this ratio is 0.6.

5. Inclination of anteroventral margin of premaxilla relative to horizontally positioned ventral margin of the jugal: vertical (0); posterodorsal (1); anterodorsal (2).

6. Ventral projection of premaxilla below ventral margin of the maxilla: absent (0); small (1); significant (2). The difference between “small” and “significant” is partially subjective and partially controlled by relative size. A line drawn from the bottom of the quadrate to the bottom of the premaxilla is below the ventral projection of the maxilla in all oviraptorids where these three points can be seen.

7. Share of premaxilla (ventral) in basal skull length: 0.10 or less (0); 0.12 or more (1). The premaxilla in the holotype is more than 0.20 of the basal skull length.

8. Pneumatization of premaxilla: absent (0); present (1).

9. Ratio of length of maxilla (in lateral view) to basal skull length: 0.40–0.69 (0); less than 0.39 (1). An extra decimal point has been added to the numbers of Osmólska et al. [10] for greater specificity.

10. Subantorbital portion of maxilla: not inset medially (0); inset medially (1).

11. Palatal shelf of maxilla with two longitudinal ridges and tooth-like ventral process: absent (0); present (1).

12.Ventral margins of maxilla and jugal: margins form straight line (0); ventral margin of maxilla slopes anteroventrally, its longitudinal axis at an angle of ca. 120° to the longitudinal axis of the jugal (1).

13. Rim around anterior and dorsal margins of antorbital fossa: well pronounced (0); poorly delimited (1). Osmólska et al. [10] referred to only the “rim along anterior and dorsal margins”, but this has been changed to “rim around the antorbital fossa” because the ventral and posterior rims (especially on the lacrimal) are also sharply defined in some specimens. The rim is well defined in the holotype of *Nemegtomaia*.

14. Antorbital fossa: bordered anteriorly by maxilla (0); bordered anteriorly by premaxilla (1).

15. Accessory maxillary fenestrae: absent (0); at least one accessory fenestra present (1).

16. Nasal along midline: longer than frontal (0); shorter than or as long as frontal (1). This needs to be measured consistently along the same axis (parallel to a line between the ventral points of the quadrate and premaxilla).

17.Nasals: separate (0); fused (1).

18. Subnarial process of nasal: long (0); short (1).

19. Shape of the narial opening: longitudinally oval (0); teardrop-shaped, slightly longer anteroposteriorly than tall (1); much longer than high (2). Although the posterior margin of the external naris is damaged, it is still clear that the external naris is significantly longer than high in the holotype of *Nemegtomaia*.

20. Nasal recesses: absent (0); present (1).

21. External naris position relative to antorbital fossa: naris and fossa widely separated anteroposteriorly (0); posterior margin of naris reaching level of fossa (1); naris overlaps anterodorsally most of the fossa (2) (modified for clarity).

22. Anteroventral margin of external naris: at the level of the maxilla (0); dorsal to maxilla (1).

23. Prefrontal: present (0); absent or fused with lacrimal (1).

24. Lacrimal shaft: not projecting outward beyond orbital plane and lateral surface of the snout (0); medial part of shaft projecting laterally to form flattened transverse bar in front of eye (1).

25. Lacrimal recesses: absent (0); present (1).

26. Ratio of length of orbit to length of antorbital fossa: 0.7–0.9 (0); 1.2 or more (1).

27. Ratio of length of parietal to length of frontal: 0.6 or less (0); 1.0 or more (1).

28. Pneumatization of skull-roof bones: absent (0); present (1).

29. Sagittal crest along interparietal contact: absent (0); present (1).

30. Supratemporal fossa: invading frontal (0); not invading frontal (1).

31. Infratemporal fenestra: dorsoventrally elongate, narrow anteroposteriorly (0); subquadrate, with anteroposterior length comparable to orbital length (1).

32. Pneumatization of squamosal: absent (0); present (1).

33.Cotylelike incision on ventrolateral margin of the squamosal (for reception of dorsal end of ascending process of the quadratojugal): absent (0); present (1).

34. Ventral ramus of jugal: dorsoventrally deep and lateromedially flattened (0); dorsoventrally shallow or rodlike (1).

35.Jugal process of postorbital: not extending ventrally below two-thirds of orbit height (0); long, extending ventrally close to base of postorbital process of jugal (1).

36.Postorbital process of jugal: less than 85^o^ between the postorbital and quadratojugal processes (0); perpendicular (85-90^o^) to ventral ramus of jugal (1); absent (2). This was rewritten from Osmólska et al. [10] to make it less ambiguous when the suborbital and quadratojugal processes are not aligned.

37. Jugal-postorbital contact: present (0); absent (1).

38. Quadratojugal process of jugal in lateral view: forked (0); not forked (1); fused with quadratojugal (2).

39. Quadratojugal-squamosal contact: absent (0); present (1).

40. Ascending (squamosal) process of quadratojugal: bordering approximately ventral half, or less, of infratemporal fenestra (0); bordering ventral two-thirds or more of infratemporal fenestra (1); absent (2).

41.Angle between ascending and jugal processes of quadratojugal: approximately 90° (0); less than 90° (1).

42.Quadrate process of quadratojugal: well developed, extending posteriorly or posteroventrally beyond posterior margin of ascending process (0); not extending beyond posterior margin of ascending process (1).

43. Dorsal part of quadrate: erect (0); bent backward (1).

44. Otic process of quadrate: articulating only with squamosal (0); articulating with squamosal and lateral wall of braincase (1).

45. Pneumatization of quadrate: absent (0); present (1).

46. Lateral accessory process on distal end of quadrate for contact with quadratojugal: absent (0); present (1).

47. Lateral cotyle for quadratojugal on quadrate: absent (0); present (1).

48. Mandibular condyles of quadrate: posterior to occipital condyle (0); in same vertical plane as occipital condyle (1); anterior to occipital condyle (2).

49. Nuchal transverse crest: pronounced (0); not pronounced (1).

50.Occiput position in relation to ventral margin of jugal-quadratojugal bar: approximately perpendicular (0); inclined anterodorsally (1).

51. Paroccipital process: directed laterally (0); directed ventrally (1).

52. Foramen magnum: smaller than or equal in size to occipital condyle (0); larger than occipital condyle (1).

53. Basal tubera: modestly pronounced (0); well developed, widely separated (1).

54. Pneumatization of basisphenoid: weak or absent (0); extensive (1).

55. Basipterygoid processes: well developed (0); strongly reduced (1); absent (2).

56. Parasphenoid rostrum: horizontal or anterodorsally directed (0); sloping anteroventrally (1).

57. Depression in periotic region: absent (0); present (1).

58. Pneumatization of periotic region: absent or weak (0); extensive (1).

59. Quadrate ramus of pterygoid: distant from braincase wall (0); overlapping braincase (1).

60. Pterygoid basal process for contact with basisphenoid: absent (0); present (1).

61. Ectopterygoid position: lateral to pterygoid (0); anterior to pterygoid (1).

62. Ectopterygoid contacts with maxilla and lacrimal: absent (0); present (1).

63. Ectopterygoid: short anteroposteriorly with hooklike jugal process (0); elongate, shaped like a Viking ship, without hooklike process (1).

64. Massive pterygoid-ectopterygoid longitudinal bar: absent (0); present (1).

65. Palate extending below cheek margin: absent (0); present (1).

66. Palatine: tetraradiate or trapezoid (0); triradiate, without jugal process (1); developed in horizontal, longitudinal, and transverse planes perpendicular to each other (2).

67. Pterygoid wing of palatine: dorsal to pterygoid (0); ventral to pterygoid (1).

68. Maxillary process of palatine: shorter than vomeral process (0); longer than vomeral process (1).

69. Vomer: distant from parasphenoid rostrum (0); approaching or in contact with parasphenoid rostrum (1).

70. Suborbital (ectopterygoid‑palatine) fenestra: well developed (0); closed or reduced (1).

71. Jaw joint: distant from midline of skull (0); close to skull midline (1).

72. Movable intramandibular joint: present (0); suppressed (1).

73. Mandibular symphysis: loose (0); tightly sutured (1); fused (2).

74. Extended symphyseal shelf at mandibular symphysis: absent (0); rudimentary (1); present (2).

75. Downturned symphyseal portion of dentary: absent (0); present (1).

76. U‑shaped mandibular symphysis: absent (0); present (1).

77. Ratio of length of retroarticular process to total mandibular length: less than 0.05 or process absent (0); ca. 0.10 (1).

78. Ratio of maximum height of mandible to mandibular length: ca. 0.2 (0); ca. 0.1 (1); 0.3–0.4 (2).

79. Ratio of height to length of external mandibular fenestra: 0.2–0.5 (0); 0.7–1.0 (1); fenestra absent (2).

80. Ratio of length of external mandibular fenestra to total mandibular length: 0.15–0.20 (0); not more than 0.10 or fenestra absent (1); 0.25 or more (2).

81.Process of surangular dividing external mandibular fenestra: absent (0); long (1); short (2) (ORDERED).

82. Co-ossification of articular with surangular: absent (0); present (1).

83. Mandibular rami in dorsal view: straight (0); laterally bowed at mid-length (1).

84. Anterodorsal margin of dentary: straight (0); concave (1).

85. Posterior margin of dentary: incised, producing two posterior processes (0); oblique (1).

86. Posterodorsal process of dentary long and shallow: present (0); absent (1).

87. Posteroventral process of dentary shallow and long, extending posteriorly at least to posterior border of external mandibular fenestra: absent (0); present (1).

88. Coronoid eminence: absent (0); present (1).

89. Posterior surangular foramen: present (0); absent (1). (Character modified).

90. Mandibular articular facet for quadrate: comprising surangular and articular (0); formed exclusively of articular (1).

91. Mandibular articular facet for quadrate: with one or two cotyles (0); convex in lateral view, transversely wide (1).

92. Position of articular facet for quadrate: below level of adjoining dorsal margin of mandibular ramus (0); above this margin (1).

93. Anterior part of prearticular: deep, approaching dorsal margin of mandible (0); shallow, straplike, not approaching dorsal mandibular margin (1).

94. Splenial: subtriangular, approaching dorsal mandibular margin (0); straplike, shallow, not approaching margin (1).

95. Mandibular adductor fossa: anteriorly delimited, occupying posterior part of mandible (0); large, anteriorly and dorsally extended, not delimited anteriorly (1).

96. Coronoid bone: well developed (0); strongly reduced (1); absent (2).

97. Premaxillary teeth: present (0); absent (1).

98. Maxillary tooth row: extends at least to level of preorbital bar (0); does not reach level of preorbital bar (1); maxillary teeth absent (2).

99. Dentary teeth: present (0); absent (1).

100. Number of cervicals (excluding cervicodorsal): not more than 10 (0); more than 10 (1).

101. Anterior articular facets of centra in anterior postaxial cervicals: not inclined or only slightly inclined (0); strongly inclined posteroventrally, almost continuous with ventral surfaces of centra (1).

102. Centra of anterior cervicals: not extending posteriorly beyond their respective neural arches (0); extending posteriorly beyond their respective neural arches (1).

103. Epipophyses on postaxial cervicals: in form of a low crest or rugosity (0); prong‑shaped (1).

104. Cervical ribs in adults: loosely attached to vertebrae (0); firmly attached (1) or fused (2).

105. Shafts of cervical ribs: longer than their respective centra (0); not longer than their respective centra (1).

106. Pleurocoels on dorsal centra: absent (0); present (1).

107.Ossified uncinate processes on dorsal ribs: absent (0); present (1).

108. Number of vertebrae included in synsacrum in adults: not more than 5 (0); 6 (1); 7–8 (2).

109. Sacral spines in adults: unfused (0); fused (1).

110. Pleurocoels on sacral centra: absent (0); present (1).

111. Transition point on caudals: absent (0); present (1).

112. Number of caudals with transverse processes: 15 or more (0); fewer than 15 (1).

113. Pleurocoels on caudal centra: absent (0); present at least in proximal part of tail (1).

114. Neural spines confined to: at least 23 proximal caudals (0); at most 16 proximal caudals (1).

115. Number of caudals: more than 35 (0); 30 or fewer (1).

116. Distal caudal prezygapophyses: overlapping less than half (0) or at least half (1) of centrum of preceding vertebra.

117. Hypapophyses in cervicodorsal vertebral region: absent (0); small (1); prominent (2).

118. Distal chevrons: deeper than long (0); longer than deep (1).

119. Ratio of length of scapula to length of humerus: 0.8–1.1 (0); 1.2 or more (1); 0.7 or less (2).

120. Acromion: projecting dorsally (0); projecting anteriorly (1); everted laterally (2).

121. Posteroventral process of coracoid: absent or short, not extending beyond glenoid diameter (0); long, posteroventrally extending beyond glenoid (1).

122. Orientation of glenoid on pectoral girdle: posteroventral (0); lateral (1).

123. Deltopectoral crest: low, its width equal to, or smaller than, shaft diameter (0); expanded, wider than shaft diameter (1).

124. Extent of deltopectoral crest (measured from humeral head to apex): about proximal third of humerus length or less (0); ca. 40%–50% of humerus length (1).

125. Shaft of ulna: straight (0); bowed, convex posteriorly (1).

126. Ratio of length of radius to length of humerus: 0.80 or less (0); 0.85 or more (1).

127. Combined lengths of manual phalanges III‑1 and III‑2: greater than length of phalanx III‑3 (0); less than or equal to length of phalanx III‑3 (1).

128. Ratio of length of metacarpal I to length of metacarpal II: 0.5 or more (0); less than 0.5 (1).

129. Proximal margin of metacarpal I in dorsal view: straight, horizontal (0); angled due to a medial extent of carpal trochlea (1).

130. Metacarpal II relative to metacarpal III: shorter (0); longer (1); subequal (within 20%) (2).

131. Ratio of length of metacarpal II to length of humerus: 0.4 or less (0); more than 0.4 (1).

132. Ratio of length of manus to length of humerus plus radius: 0.50–0.65 (0); more than 0.65 (1); less than 0.50 (2). Ratio is 0.80 in Oviraptor, which is therefore coded as “1”.

133. Ratio of length of manus to length of femur: 0.3–0.6 (0); more than 0.7 (1).

134. Ratio of length of humerus to length of femur: 0.50–0.69 (0); 0.70 or more (1). Character modified from Osmólska et al. [10].

135. Dorsal margins of opposite iliac blades: well separated from each other (0); close to or contacting each other along their medial sections (1).

136. Dorsal margin of ilium along central portion of blade: straight (0); arched (1).

137. Preacetabular process of ilium relative to postacetabular process (lengths measured from center of acetabulum): shorter or equal (0); longer (1).

138. Preacetabular process: not expanded or weakly expanded ventrally below level of dorsal acetabular margin (0); expanded ventrally well below level of dorsal acetabular margin (1).

139. Morphology of ventral margin of preacetabular process: cuppedicus fossa absent, margin transversely narrow (0); cuppedicus fossa or a wide shelf present (1); margin flat, wide at least close to pubic peduncle (2).

140. Anteroventral extension of preacetabular process: absent (0); with rounded tip (1); hooklike (2).

141. Distal end of postacetabular process: truncated or broadly rounded (0); narrowed or acuminate (1).

142. Anteroposterior length of pubic peduncle: about same as that of ischial peduncle (0); distinctly greater than that of ischial peduncle (1).

143. Dorsoventral extension of pubic peduncle: level with ischial peduncle (0); deeper than ischial peduncle (1).

144. Ratio of length of ilium to length of femur: 0.50–0.79 (0); 0.80 or more (1). Character modified from Osmólska et al. [10] by adding an extra decimal position.

145. Pelvis: propubic (0); mesopubic (1); opisthopubic (2).

146. Pubic shaft: straight (0); concave anteriorly (1).

147. Pubic foot: anterior and posterior processes about equally long (0); anterior process absent or shorter than posterior process (1); anterior process longer than posterior process (2).

148. Posterior margin of ischial shaft: straight, or almost straight (0); distinctly concave (1).

149. Greater trochanter of femur: weakly separated, or not separated, from femoral head (0); distinctly separated from femoral head (1).

150. anterior and greater trochanters: separated (0); contacting (1).

151. Dorsal extremity of anterior trochanter: well below greater trochanter (0); about level with greater trochanter (1).

152. Fourth trochanter: well developed (0); weakly developed or absent (1).

153. Adductor fossa and associated anteromedial crest on distal femur: weak or absent (0); well developed (1).

154. Distal projection of fibular condyle of femur beyond tibial condyle: absent (0); present (1).

155. Ascending process of astragalus: as tall as it is wide across base (0); taller than wide (1).

156. Distal tarsals: not fused with metatarsus (0); fused with metatarsus (1).

157. Proximal co-ossification of metatarsals II–IV: absent (0); present (1).

158. Arctometatarsus: absent (0); present (1).

159. Length of metatarsal I constituting: more than 50% of metatarsal II length (0); less than 50% of metatarsal II length (1); metatarsal I absent (2).

160. Ratio of maximum length of metatarsus to length of femur: 0.4–0.6 (0); ca. 0.3 (1); 0.7–0.8 (2).

161. Crenulated tomial margin of premaxilla: absent (0); present (1).

162. Frontals flat or weakly arched, not strongly projecting above orbit in lateral view (0) or frontals strongly arched, projecting well above orbit in lateral view to contribute to nasal-frontal crest (1).

163. Exoccipital short, weakly projecting (0) or exoccipital strongly projects ventrally beyond squamosal in lateral view, approaching ventral end of the quadrate (1).

164. Dentary posterodorsal ramus straight or weakly curved (0) or strongly bowed dorsally (1). Oviraptor was recoded from “?” to “0” on basis of 40.

165. Dentary symphyseal ventral process absent (0) or prominent symphyseal process present on posteroventral surface of symphysis (1).

166. Dentary anteroventral margin straight or weakly downturned (0) or strongly downturned in lateral view (1).

167. Lateral surface of dentary smooth (0); bearing a deep fossa, sometimes with associated pneumatopore (1).

168. Angular contributes extensively to the border of the external mandibular fenestra (0); angular largely excluded by surangular (1).

169. Surangular with an anteroposteriorly elongate flange on the ventral edge: absent (0); present (1)

170. External mandibular fenestra elongate (0) or height of external mandibular fenestra subequal to length (1).

171. Dentary contribution to EMF no more than 50 per cent length of dentary (0); exceeds 50 per cent length of dentary (1).

172. Metacarpal I expanded ventrally to cover ventral surface of metacarpal II: absent (0); present (1).

173. Unguals of manual digits II and III: strongly curved (0); weakly curved (1).

174. Manus I-1 slender (0); more robust than II (1); more than 200 per cent diameter of II-1 (2). ORDERED

175. Manual phalanx III-3 longer than phalanx III-2 (0) or length of phalanx III-3 does not exceed length of III-2 (1).

176. Manual phalanx II-2 longer than II-1(0) or manual phalanx II-2 does not exceed the length of preceding phalanx (1).

177. Manual digit II elongate, with combined lengths of manual phalanges II-1 and II-2 subequal to or longer than metacarpal II (0) or combined lengths of II-1 and II-2 subequal to MC II (1) or combined lengths of II-1 and II-2 shorter than MC II (2) ORDERED.

178. Ischium strongly bent posteriorly at midshaft, distal end forms an angle of at least 60º with proximal end: absent (0); present (1).

179. Metatarsus elongate (0); short, length does not exceed 300 per cent of proximal width (1).

180. Ilium tall (0); low and anteroposteriorly elongate, height less than 25 per cent of length (1).

181. Anterior blade of ilium shallower than posterior blade: absent (0); present (1).

182. Calcaneum excludes astragalus from reaching lateral margin of tarsus (0); small process of astragalus protrudes through a circular opening in edge of calcaneum to reach lateral margin of tarsus (1).
