# Supplementary material for: New Specimens of Nemegtomaia from the Baruungoyot and Nemegt Formations (Late Cretaceous) of Mongolia
Source: PLoS One. 2012 Feb 8;7(2):e31330. doi: 10.1371/journal.pone.0031330 (PMC3275628; doi:10.1371/journal.pone.0031330)
Supplement: Table S3 — Character-taxon matrix. (DOC) [file pone.0031330.s003.doc]

**Table S3**

**Character-taxon matrix.**

*Herrerasaurus ischigualastensis*

00000000000000000000000000000000000000000000000000000000000000000000000000000000000000000000000000000010?0000000000100?0000101??0000000000000000100000000000000000000?000?000011001010

*Velociraptor mongoliensis*

000100000000101001010010100010000000001000000001000010000000000000000000000000010000110000000000010010110111101101011101-110101111011100001111112010011100010011000000000?000000001100

*Archaeopteryx lithographica*

000?10000000101101001100010001?10102110?0?1??0020????00????0000001?1???100001121000011-0100000020100??0101?00?1101100121110011111210110110101110201-1?0100?1101200?-000?0?0?000000010?

*Avimimus portentosus*

??????????0?????1110??????1011-??1-2121201??00?101111?1???????????????0?21011???01?????11000???20?11000110?110??0???2?02100111?1?????00000?010?10020101001111122100010000?0???????00??

*Caudipteryx zoui*

10?110?01??0?1110?0?11?01?10???0110001110100??????????????????0????????0??111??2???0001??????11?0210?????010????0?1??10???1011?1020100?001220110?021100???1000121??0???0??0?00?001000?

*Chirostenotes pergracilis*

?????????01?010??????1????????????????????????????110?1?1??0??????????11220110020111001111111112?21????0?1?111??1???1???0??????0???????11???110??021???1?1?0?110???00?1010000001?10000

*Gigantoraptor erlianensis*

??????????????????????????????????????????????????????????????????????1121111202011100111111????1?1???????????0010?0??00?00000?11?1??1???????????1??1111111000?0???0001010000?????????

*Nomingia gobiensis*

??????????????????????????????????????????????????????????????????????????????????????????????????????0??1?0010011112?????????????????1111110101100111?1111??????????????????????????1

*Richenia mongoliensis*

11122?1111110111???1211111111?111101011100111111?1111121?11111111211111111111212?0110011111111121211110011?1010010102?12101000???????01101121101????111111??????1101?001011????????001

*Conchoraptor gracilis*

10121111111101111121211111111111110001110?1111111?111121111111111211111111111212?0110011111111121211110111?20100101011121011110012111010002100011121111111100010101??????0??110???1???

*Ingenia yanshini*

102201111??????11?2????11?????1?1100011110111111???111??11?1??111?1???11111112121011001111111112121?110011?20100101010121011000012020010002100011121111111100010?01-?1---1-11211101111

*Citipati osmolskae*

11?222111111011111112111111?111?1111011111??????111?1??11?1?11111????1?1111112121011001111111111121???????1??????????????????1??????????????????????????????????11011001011?01010?0??1

*Citipati n.sp.*

11222211111101111111211111111111111101111111111111111121111111111211111111111212101100111111111?1211110111?101001010210210101101121110100121100111211111111000101101?001011?0000010???

*Khaan mckennai*

10?2111?11?1011?11212111111?111?1100011100?????1111???????1?????1??????1?111?212101100111111??1?1211?101111???00??1????210??1101?20110?000?10001?1?11?1?????00101001100100100100001111

*Machairasaurus leptonychus*

?????????????????????????????????????????????????????????????????????????1?1????????????????????????????????????????????????1?0012?????????????????????????????????????????111110?????

*Heyuannia huangi*

?????????????????????????????????1????????????????????????????????????????1??2121??1001???????????11???21?12????1?????121?1101001212?0??0??110??11?1???????????????1?1?1011112?110101?

*Nemegtomaia barsboldi*

1122021111110111112121111111111?110001111011?11111111???????1111121111111111121211110011111111121211110211?201??????1?12??11010012??00100021100111??111?????????10111101011?12?11??111

*Oviraptor philoceratops*

11??????1??1?11?????21????????1??1000???0???????????????????????1????????11?12122??10011??11??1??21???0??1????????????1???101101?211?????????????????????????????0?0?001001000000??0?1

*Incisivosaurus gautheri*

?0100010110000100?0110?011001?00?0000?10011????201111111100100001110000?2101?002??000111????????010?????????????????????????????????????????????????????????????0110000?10????????????
